# 1,4-Bis(2-nitro­phen­oxy)butane

**DOI:** 10.1107/S1600536809048909

**Published:** 2009-11-25

**Authors:** Perla Elizondo, Cecilia Rodríguez de Barbarín, Blanca Nájera, Nancy Pérez

**Affiliations:** a División de Estudios de Posgrado, Facultad de Ciencias Químicas, Universidad Autónoma de Nuevo León, AP 1864, 64570 Monterrey, NL, Mexico

## Abstract

The asymmetric unit of the title compound, C_16_H_16_N_2_O_6_, contains one-half mol­ecule, the mid-point of the central C—C bond being located on a crystallographic inversion center. The crystal structure shows weak inter­actions between the O atoms of the nitro groups and two different C—H groups of the benzene rings. The extended weak hydrogen-bond formation, involving the NO_2_ groups, generates an infinite three-dimensional network.

## Related literature

For related structures, see: Han & Zhen (2005 ▶); Naz *et al.* (2007 ▶); Zhang *et al.* (2007 ▶). For recent examples of complexes with macrocyclic ligands, including diether subunits, see: Fernández *et al.* (2008 ▶); Platas-Iglesias *et al.* (2005 ▶); Tas *et al.* (2006 ▶).
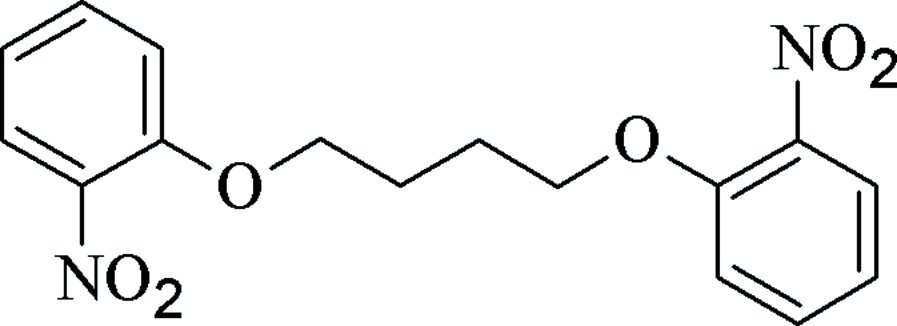


## Experimental

### 

#### Crystal data

C_16_H_16_N_2_O_6_
*M* *_r_* = 332.31
Monoclinic, 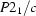

*a* = 7.7977 (8) Å
*b* = 13.888 (2) Å
*c* = 7.6729 (8) Å
β = 110.866 (6)°
*V* = 776.4 (2) Å^3^
*Z* = 2
Mo *K*α radiation
μ = 0.11 mm^−1^
*T* = 296 K
0.7 × 0.6 × 0.4 mm

#### Data collection

Bruker P4 diffractometer
Absorption correction: none
3850 measured reflections
2256 independent reflections
1752 reflections with *I* > 2σ(*I*)
*R*_int_ = 0.030
3 standard reflections every 97 reflections intensity decay: 2.3%

#### Refinement

*R*[*F* ^2^ > 2σ(*F* ^2^)] = 0.048
*wR*(*F* ^2^) = 0.130
*S* = 1.06
2256 reflections
110 parameters
H-atom parameters constrained
Δρ_max_ = 0.22 e Å^−3^
Δρ_min_ = −0.19 e Å^−3^

### 

Data collection: *XSCANS* (Siemens, 1996 ▶); cell refinement: *XSCANS*; data reduction: *SHELXTL-Plus* (Sheldrick, 2008 ▶); program(s) used to solve structure: *SHELXTL-Plus*; program(s) used to refine structure: *SHELXTL-Plus*; molecular graphics: *SHELXTL-Plus* and *Mercury* (Macrae *et al.*, 2006 ▶); software used to prepare material for publication: *SHELXTL-Plus*.

## Supplementary Material

Crystal structure: contains datablocks I, global. DOI: 10.1107/S1600536809048909/im2156sup1.cif

Structure factors: contains datablocks I. DOI: 10.1107/S1600536809048909/im2156Isup2.hkl

Additional supplementary materials:  crystallographic information; 3D view; checkCIF report

## Figures and Tables

**Table 1:** Hydrogen-bond geometry (Å, °)

*D*—H⋯*A*	*D*—H	H⋯*A*	*D*⋯*A*	*D*—H⋯*A*
C3—H3*A*⋯O1^i^	0.93	2.63	3.284 (2)	128
C5—H5*A*⋯O2^ii^	0.93	2.58	3.476 (2)	163

Symmetry codes: (i) 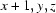; (ii) 
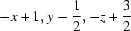
; (iii) 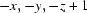.

**Fig. 1.**
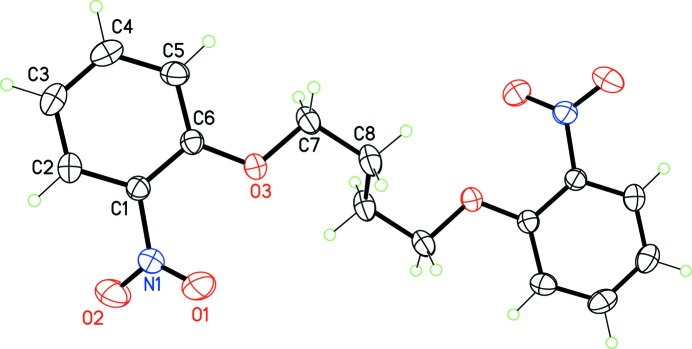
Molecular structure of (I). Displacement ellipsoids are drawn at the 30% probability level and H atoms are shown as small spheres of arbitrary radii. Atoms not labelled are related to the asymmetric unit by symmetry code -x, -y, -z + 1.

**Fig. 2.**
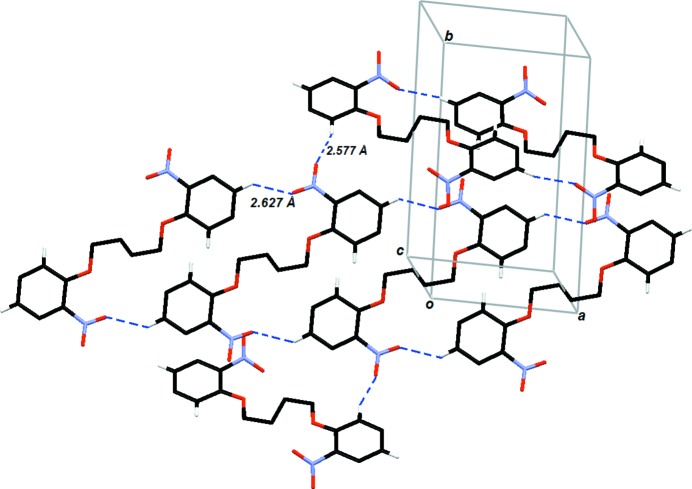
Molecular packing structure of (I) showing week interactions between O of the nitro groups and H atoms of two different C—H groups of the benzene rings (dashed bonds). H atoms not involved in this network have been omitted.

## supplementary crystallographic information

### Comment

The title compound (I) has been synthesized as a chemical precursor of a variety of acyclic and macrocyclic multidentate ligands and metal complexes.

Related compounds have been reported (Zhang *et al.*, 2007; Naz *et al.*, 2007 and Han & Zhen, 2005). Similar cyclic and macrocyclic ligands to (I), have been reported (Fernández *et al.*, 2008; Tas *et al.*, 2006 and Platas-Iglesias *et al.*, 2005).

Compound (I) crystallizes with the molecule being situated on a crystallographic inversion center that is localized at the midpoint of the C8—C8^i^ bond [symmetry code: (i) -*x*, -*y*, -*z* + 1] (Fig. 1). As a consequence of the centrosymmetric nature of the molecule a dihedral angle of 0° is observed between the benzene rings in (I). The torsion angle between a benzene ring and the corresponding nitro group is 38.5 (1)°. The conformation of the central chain is described by torsion angles, C6—O3—C7—C8, -178.2 (1)°, O3—C7—C8—C8^i^, 62.6 (2)° and C7—C8—C8^i^—C7^i^, constrained by symmetry to 180.0°. This *trans-gauche-trans* conformation stabilized in the solid state for (I) is less common than the *all-trans* conformation that is generally found in aliphatic systems. This molecular conformation is stabilized by weak intramolecular hydrogen bonds involving O3 and a symmetry related C—H group (O3···H8B 2.900 (2) Å). Nevertheless, O atoms in (I) may coordinate to a metal center as a chelating ligand after changing the conformation of this potential ligand. These observations suggest that (I) is a highly flexible molecule, with an almost free rotation about all σ bonds.

In addition, the crystal structure shows weak interactions between oxygen atoms of the nitro groups and two different C—H groups of benzene rings (O1···H3A 2.627 and O2···H5A 2.577 Å) as shown in Fig 2. The extended weak H bond formation, using the NO_2_ groups, produces an infinite three-dimensional network of the title compound.

### Experimental

*o*-Nitrophenol (23.90 g) in hot DMF (25.0 ml) was treated with potassium carbonate (11.90 g), added slowly in portions. The solution was gently boiled and 1,4-dibromobutane (8.40 ml) was added during 30 min. Gentle reflux was mantained for another 2 h. Then solvent (15.0 ml) was destilled from the mixture and the remaining mixture was poured into water (250 ml). The granular yellow solid was filtered off, washed with dilute aqueous sodium hydroxide solution and water, then dried (23.8 g). *M*.p. 442–443 K, yield 81%.

Suitable crystals were obtained as colorless blocks from acetonitrile solution by slow evaporation of the solvent at 298 K. The solid was characterized by IR (KBr disc), ^1^H-NMR and elemental analysis, which are in agreement with the X-ray structure.

### Refinement

Hydrogen atoms bonded to C atoms were included in calculated positions and refined using the riding method, with C—H distances constrained to 0.93 (aromatic CH) and 0.97 Å (methylene CH_2_) and *U*_iso_(H) = 1.2*U*_eq_(carrier C).

### Crystal data

**Table:**

C_16_H_16_N_2_O_6_	*F*(000) = 348
*M**_r_* = 332.31	*D*_x_ = 1.421 Mg m^−^^3^
Monoclinic, *P*2_1_/*c*	Melting point = 442–443 K
Hall symbol: -P 2ybc	Mo *K*α radiation, λ = 0.71073 Å
*a* = 7.7977 (8) Å	Cell parameters from 85 reflections
*b* = 13.888 (2) Å	θ = 5.2–12.4°
*c* = 7.6729 (8) Å	µ = 0.11 mm^−^^1^
β = 110.866 (6)°	*T* = 296 K
*V* = 776.4 (2) Å^3^	Block, colorless
*Z* = 2	0.7 × 0.6 × 0.4 mm

### Data collection

**Table:**

Bruker P4 diffractometer	*R*_int_ = 0.030
Radiation source: fine-focus sealed tube	θ_max_ = 30.0°, θ_min_ = 2.9°
graphite	*h* = −10→10
ω scan	*k* = −1→19
3850 measured reflections	*l* = −10→5
2256 independent reflections	3 standard reflections every 97 reflections
1752 reflections with *I* > 2σ(*I*)	intensity decay: 2.3%

### Refinement

**Table:**

Refinement on *F*^2^	Primary atom site location: structure-invariant direct methods
Least-squares matrix: full	Secondary atom site location: difference Fourier map
*R*[*F*^2^ > 2σ(*F*^2^)] = 0.048	Hydrogen site location: inferred from neighbouring sites
*wR*(*F*^2^) = 0.130	H-atom parameters constrained
*S* = 1.06	*w* = 1/[σ^2^(*F*_o_^2^) + (0.0452*P*)^2^ + 0.1839*P*] where *P* = (*F*_o_^2^ + 2*F*_c_^2^)/3
2256 reflections	(Δ/σ)_max_ < 0.001
110 parameters	Δρ_max_ = 0.22 e Å^−^^3^
0 restraints	Δρ_min_ = −0.19 e Å^−^^3^

### Special details

**Table:**

Experimental. Experimental absorption correction were not applied because the molecule is purely organic, and no better structure refinement was obtained.
Geometry. All e.s.d.'s (except the e.s.d. in the dihedral angle between two l.s. planes) are estimated using the full covariance matrix. The cell e.s.d.'s are taken into account individually in the estimation of e.s.d.'s in distances, angles and torsion angles; correlations between e.s.d.'s in cell parameters are only used when they are defined by crystal symmetry. An approximate (isotropic) treatment of cell e.s.d.'s is used for estimating e.s.d.'s involving l.s. planes.
Refinement. Refinement of *F*^2^ against ALL reflections. The weighted *R*-factor *wR* and goodness of fit *S* are based on *F*^2^, conventional *R*-factors *R* are based on *F*, with *F* set to zero for negative *F*^2^. The threshold expression of *F*^2^ > σ(*F*^2^) is used only for calculating *R*-factors(gt) *etc*. and is not relevant to the choice of reflections for refinement. *R*-factors based on *F*^2^ are statistically about twice as large as those based on *F*, and *R*- factors based on ALL data will be even larger.

### Fractional atomic coordinates and isotropic or equivalent isotropic displacement parameters (Å^2^)

**Table:**

	*x*	*y*	*z*	*U*_iso_*/*U*_eq_	
O1	0.19481 (19)	0.21566 (10)	0.9250 (3)	0.0923 (5)	
O2	0.32880 (19)	0.33983 (9)	0.8740 (2)	0.0827 (4)	
O3	0.28310 (12)	0.07009 (6)	0.74649 (13)	0.0474 (3)	
N1	0.32195 (16)	0.25370 (9)	0.89929 (17)	0.0506 (3)	
C1	0.47947 (16)	0.19562 (9)	0.90340 (16)	0.0392 (3)	
C2	0.65074 (18)	0.23662 (10)	0.98583 (19)	0.0487 (3)	
H2A	0.6618	0.2979	1.0376	0.058*	
C3	0.80442 (19)	0.18694 (12)	0.9912 (2)	0.0575 (4)	
H3A	0.9205	0.2136	1.0478	0.069*	
C4	0.7837 (2)	0.09688 (12)	0.9114 (2)	0.0579 (4)	
H4A	0.8874	0.0634	0.9126	0.070*	
C5	0.61331 (19)	0.05505 (10)	0.8296 (2)	0.0497 (3)	
H5A	0.6038	−0.0060	0.7772	0.060*	
C6	0.45534 (16)	0.10362 (9)	0.82495 (16)	0.0388 (3)	
C7	0.2563 (2)	−0.01938 (9)	0.6464 (2)	0.0495 (3)	
H7A	0.3192	−0.0712	0.7294	0.059*	
H7B	0.3044	−0.0151	0.5462	0.059*	
C8	0.0531 (2)	−0.03792 (10)	0.5688 (2)	0.0534 (4)	
H8A	0.0081	−0.0418	0.6699	0.064*	
H8B	0.0315	−0.0991	0.5063	0.064*	

### Atomic displacement parameters (Å^2^)

**Table:**

	*U*^11^	*U*^22^	*U*^33^	*U*^12^	*U*^13^	*U*^23^
O1	0.0717 (8)	0.0766 (9)	0.1558 (14)	−0.0089 (7)	0.0739 (9)	−0.0235 (9)
O2	0.0880 (9)	0.0461 (6)	0.1202 (12)	0.0135 (6)	0.0448 (8)	0.0006 (7)
O3	0.0423 (5)	0.0429 (5)	0.0527 (5)	−0.0051 (4)	0.0117 (4)	−0.0121 (4)
N1	0.0481 (6)	0.0500 (6)	0.0554 (7)	−0.0005 (5)	0.0205 (5)	−0.0119 (5)
C1	0.0392 (6)	0.0400 (6)	0.0386 (6)	−0.0007 (4)	0.0142 (5)	−0.0014 (5)
C2	0.0467 (7)	0.0481 (7)	0.0479 (7)	−0.0098 (5)	0.0125 (5)	−0.0025 (5)
C3	0.0388 (6)	0.0690 (9)	0.0584 (8)	−0.0073 (6)	0.0098 (6)	0.0055 (7)
C4	0.0425 (7)	0.0699 (9)	0.0606 (9)	0.0146 (6)	0.0174 (6)	0.0126 (7)
C5	0.0506 (7)	0.0459 (7)	0.0510 (7)	0.0100 (5)	0.0160 (6)	0.0012 (6)
C6	0.0393 (6)	0.0388 (6)	0.0365 (5)	−0.0013 (4)	0.0111 (4)	0.0003 (4)
C7	0.0574 (8)	0.0354 (6)	0.0501 (7)	−0.0045 (5)	0.0122 (6)	−0.0054 (5)
C8	0.0603 (8)	0.0385 (6)	0.0520 (8)	−0.0123 (6)	0.0085 (6)	0.0006 (5)

### Geometric parameters (Å, °)

**Table:**

O1—N1	1.2000 (16)	C4—C5	1.380 (2)
O2—N1	1.2159 (17)	C4—H4A	0.9300
O3—C6	1.3443 (15)	C5—C6	1.3937 (18)
O3—C7	1.4363 (15)	C5—H5A	0.9300
N1—C1	1.4604 (16)	C7—C8	1.504 (2)
C1—C2	1.3807 (17)	C7—H7A	0.9700
C1—C6	1.3962 (17)	C7—H7B	0.9700
C2—C3	1.371 (2)	C8—C8^i^	1.512 (3)
C2—H2A	0.9300	C8—H8A	0.9600
C3—C4	1.377 (2)	C8—H8B	0.9601
C3—H3A	0.9300		
			
C6—O3—C7	118.09 (10)	C4—C5—H5A	119.8
O1—N1—O2	123.04 (14)	C6—C5—H5A	119.8
O1—N1—C1	119.40 (13)	O3—C6—C5	125.22 (12)
O2—N1—C1	117.54 (12)	O3—C6—C1	118.02 (11)
C2—C1—C6	122.32 (11)	C5—C6—C1	116.73 (11)
C2—C1—N1	116.81 (11)	O3—C7—C8	106.96 (11)
C6—C1—N1	120.86 (11)	O3—C7—H7A	110.3
C3—C2—C1	119.93 (13)	C8—C7—H7A	110.3
C3—C2—H2A	120.0	O3—C7—H7B	110.3
C1—C2—H2A	120.0	C8—C7—H7B	110.3
C2—C3—C4	118.77 (13)	H7A—C7—H7B	108.6
C2—C3—H3A	120.6	C7—C8—C8^i^	113.25 (14)
C4—C3—H3A	120.6	C7—C8—H8A	109.1
C3—C4—C5	121.74 (13)	C8^i^—C8—H8A	109.7
C3—C4—H4A	119.1	C7—C8—H8B	108.7
C5—C4—H4A	119.1	C8^i^—C8—H8B	108.2
C4—C5—C6	120.48 (13)	H8A—C8—H8B	107.7
			
O1—N1—C1—C2	−141.19 (16)	C7—O3—C6—C1	172.71 (11)
O2—N1—C1—C2	37.07 (18)	C4—C5—C6—O3	178.93 (13)
O1—N1—C1—C6	39.59 (19)	C4—C5—C6—C1	1.0 (2)
O2—N1—C1—C6	−142.15 (14)	C2—C1—C6—O3	−179.48 (12)
C6—C1—C2—C3	0.5 (2)	N1—C1—C6—O3	−0.30 (17)
N1—C1—C2—C3	−178.69 (13)	C2—C1—C6—C5	−1.43 (18)
C1—C2—C3—C4	0.8 (2)	N1—C1—C6—C5	177.75 (12)
C2—C3—C4—C5	−1.2 (2)	C6—O3—C7—C8	−178.18 (11)
C3—C4—C5—C6	0.2 (2)	O3—C7—C8—C8^i^	62.6 (2)
C7—O3—C6—C5	−5.15 (19)		

Symmetry codes: (i) −*x*, −*y*, −*z*+1.

### Hydrogen-bond geometry (Å, °)

**Table:**

*D*—H···*A*	*D*—H	H···*A*	*D*···*A*	*D*—H···*A*
C3—H3A···O1^ii^	0.93	2.63	3.284 (2)	128
C5—H5A···O2^iii^	0.93	2.58	3.476 (2)	163
C8—H8B···O3^i^	0.96	2.56	2.900 (2)	101

Symmetry codes: (ii) *x*+1, *y*, *z*; (iii) −*x*+1, *y*−1/2, −*z*+3/2; (i) −*x*, −*y*, −*z*+1.
